# Circulating exosomal microRNAs reveal the mechanism of Fructus Meliae Toosendan-induced liver injury in mice

**DOI:** 10.1038/s41598-018-21113-6

**Published:** 2018-02-12

**Authors:** Jie Zheng, Lingqi Yu, Wen Chen, Xiaoyan Lu, Xiaohui Fan

**Affiliations:** Pharmaceutical Informatics Institute, College of Pharmaceutical Sciences, Zhejiang University, Hangzhou, 310058 China

## Abstract

The toxicological mechanisms of liver injury caused by most traditional Chinese medicine (TCM) remain largely unknown. Due to the unique features, exosomal microRNAs (miRNAs) are currently attracting major interests to provide further insights into toxicological mechanisms. Thus, taking Fructus Meliae Toosendan as an example of hepatoxic TCM, this study aimed to elucidate its hepatotoxicity mechanisms through profiling miRNAs in circulating exosomes of Fructus Meliae Toosendan water extract (FMT)-exposed mice. Biological pathway analysis of the 64 differentially expressed exosomal miRNAs (DEMs) showed that hepatic dysfunction induced by FMT likely related to apoptosis, mitochondrial dysfunction, and cell cycle dysregulation. Integrated analysis of serum exosomal DEMs and hepatic differentially expressed mRNAs further enriched oxidative stress and apoptosis related pathways. *In vitro* validation studies for omics results suggested that FMT-induced DNA damage was mediated by generating intracellular reactive oxygen species, leading to cell apoptosis through p53-dependent mitochondrial damage and S-phase arrest. Nrf2-mediated antioxidant response was activated to protect liver cells. Moreover, serum exosomal miR-370-3p, the most down-regulated miRNA involving in these pathways, might be the momentous event in aggravating cytotoxic effect of FMT by elevating p21 and Cyclin E. In conclusion, circulating exosomal miRNAs profiling could contribute to deepen the understanding of TCM-induced hepatotoxicity.

## Introduction

With the increasing application of traditional Chinese medicine (TCM) in clinic, TCM-induced liver injury (TCM-ILI) becomes a frequent cause of hepatic dysfunction^1^, which accounts for approximately 19 to 63% cases of all instances of hepatic injury in Asian countries^2,3^. Furthermore, the manifestations of TCM-ILI are highly variable, ranging from asymptomatic elevation of liver enzymes to fatal hepatic failure. It is urgent to explore the pathophysiologic mechanisms of TCM-ILI for the safe use of TCM. However, due to intrinsic complexity of TCM, understanding the mechanisms of TCM-ILI is quite difficult.

Fructus Meliae Toosendan (FMT), a typical hepatotoxic TCM, is the mature fruit from *Melia Toosendan Sieb*. Et.Zucc. (ChuanLianZi in Chinese) and it has been widely used in China and Korea for a long time to treat ascariasis, stomach ache, cholecystitis, gastritis, cholelithiasis, and mastitis^4^. Although FMT-induced liver injury has been reported frequently in recent years^5,6^, the hepatotoxicity mechanisms of FMT are still poorly clarified, which greatly limits the safe application of this medicinal TCM. To address this problem, many efforts have been made based on the isolation of single component of FMT, such as triterpenoids, steroids, and limonoids^7–9^. However, due to the clinical application form of FMT containing multiple components, it is still an open question whether specific component is solely responsible for FMT-induced liver injury. Like most other TCM, decoction is the most commonly used form of FMT in clinic. Therefore, investigating the hepatotoxicity mechanisms of FMT water extract is more meaningful than detecting those of single component. In our previous study^10^, we investigated the hepatotoxicity mechanisms of FMT water extract by an integrated analysis of microRNAs (miRNAs) and mRNAs expression profiles in the liver. Oxidative stress is shown to play a vital role in FMT water extract-induced liver injury (FMT-ILI). However, further clarification of molecular and cellular toxicological mechanisms of FMT-ILI is needed at the molecular level.

Recently, exosomes have drawn considerable attentions for providing sensitive and detailed insights into many diseases. Exosomes are small membrane vesicles that range in size between 40 and 100 nm^11^. Various types of cells can release exosomes into blood or other body fluids. The cargoes in exosomes, such as RNAs (mRNAs and miRNAs), proteins, and surface molecules^12^, reflect the specific stresses that induce their formation and release^13,14^. Once secreted from the parental cells, exosomes can act on a number of target cells in the vicinity of the origin through a juxtacrine manner or deliver long-range signals at distant sites^13^, which provide a novel mechanism for cell-to-cell communication. Therefore, exosomes could participate in pathophysiological processes of disease and be used as biomarkers to assess the tissue functions^15^. Proof-of-concept studies from several groups have confirmed that changes in liver physiology and onset of liver damage would trigger perturbations in circulating exosomes. Wetmore *et al*. found that liver-specific mRNAs (e.g., albumin, fibrinogen B β-polypeptide, and haptoglobin) were packed into exosomes and released into blood in response to liver damage^16^. Additionally, some exosomal proteins in urine (e.g., CD26, CD81, SLC3A1, and CD10) were proposed to distinguish diverse liver damage as well^17^. These studies highlighted the potential utility of cargoes in circulating exosomes as characteristics for liver injury.

It is not surprising to observe increasing toxicological studies focused on the role of circulating exosomal miRNAs in liver diseases and their diagnostic and therapeutic potential for several advantages of these miRNAs, including remarkable stability, resistance to degradation, and reflection of pathophysiological processes^18^. For instance, exosomal miR-122 and miR-155 increased during acute liver injury and were even earlier markers of hepatocellular destruction than serum transaminases^18^. Interestingly, while elevated levels of miR-122 and miR-155 were detected in circulating exosomes in mice with alcohol- or lipopolysaccharide-induced liver injury, these exosomal miRNAs remained unchanged for acetaminophen-induced hepatotoxicity. It was suggested that miRNAs compartment distribution pattern differs widely depending on the etiology^18^. More importantly, it has been demonstrated that circulating exosomal miRNAs may provide useful information involved in the mechanisms of drug-induced liver injury (DILI). For example, Momen *et al*. reported that the level of miR-122 in serum exosomes was elevated when alcohol-induced liver injury occurred. Then the functional study indicated that the liver-specific miRNA-122 was transferred via exosomes from hepatocytes to monocytes and reprogramed monocytes inducing sensitization to inflammation in liver injury^19^.

**Figure 1:**
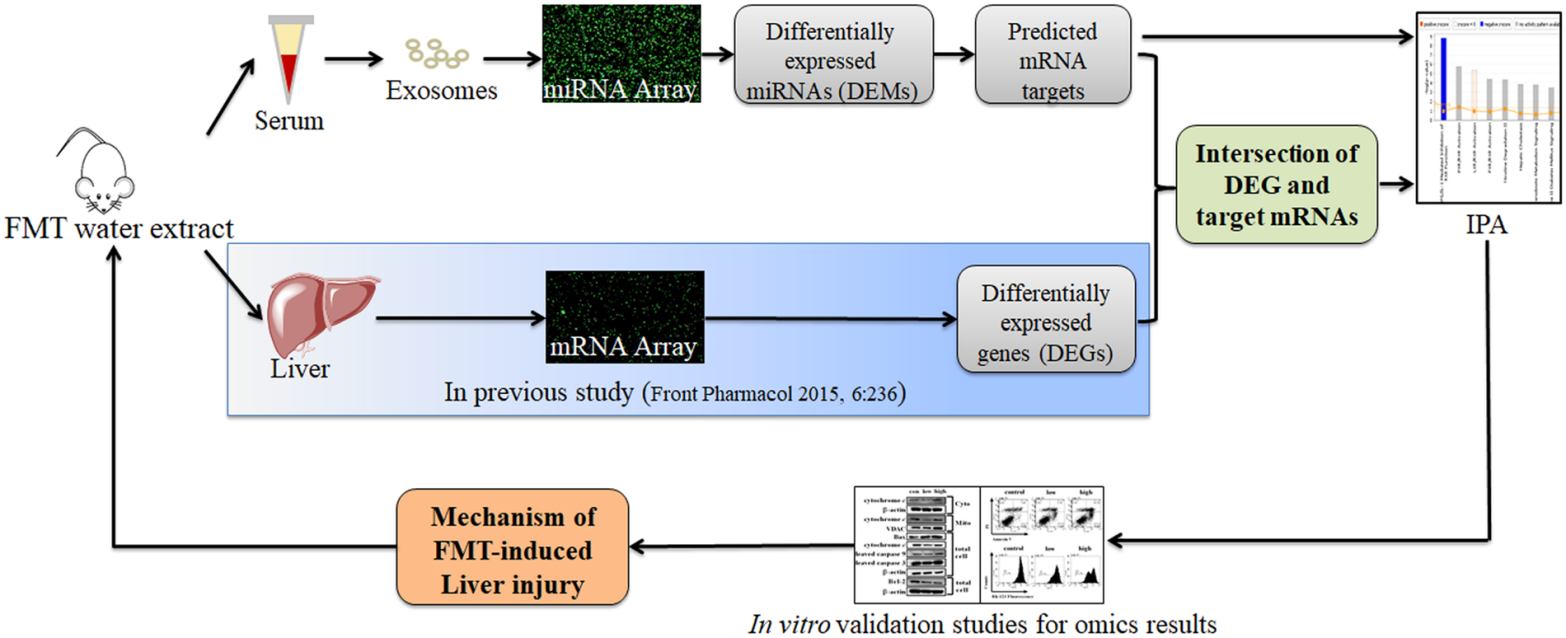
Experimental design of this study.

Thus, the objective of this study is to clarify the exact molecular and cellular mechanisms of FMT-ILI by microarray analysis of serum exosomal miRNAs profiles in FMT water extract-exposed mice. Then the functions of the altered miRNAs were assessed by Ingenuity Pathway Analysis (IPA). Since liver is the main target organ of FMT-ILI demonstrated by our previous study^10^, the integrated analysis of serum exosomal miRNAs and hepatic mRNAs expression profiles was further performed. A series of confirmatory experiments *in vitro* were taken to validate the possible molecule mechanisms of FMT-ILI. The experimental design of this study was shown in Fig. 1. In the following sections, FMT represents the water extract of FMT for short.

## Results

### Identification and characterization of serum exosomes

Similar to the results in our previously published study^10^, exposure to FMT induced liver injury in mice with significant increases in serum ALT and AST activities and induction of hydropic degeneration of hepatocytes (data not shown).

**Figure 2:**
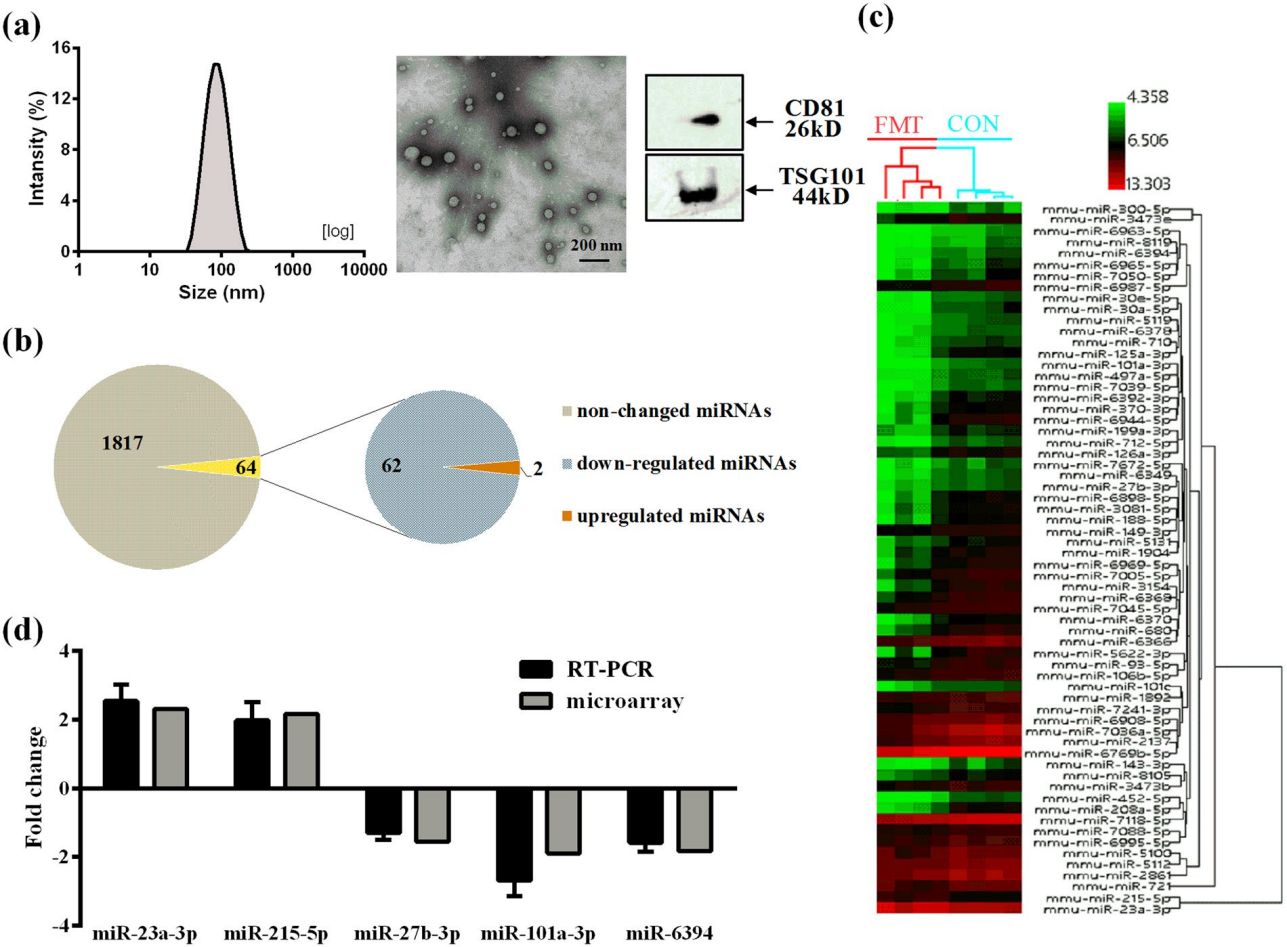
(**a**) The size distribution of the serum exosomes was determined using dynamic light scattering (left). Transmission electron micrograph of serum exosomes (middle). The scale bar is 200 nm. The expressions of exosomal marker proteins TSG101 and CD81 were determined using western blot (right). Full-length western blot images in (**a**) are presented in Supplementary Fig. S1. (**b**) The differentially expressed miRNAs in serum exosomes with the administration of FMT. (**c**) HCA showed two main branches with the DEMs in serum exosomes. (**d**) Real-time quantitative PCR was applied to validate the results of microarray analysis. Gray bars represent microarray data. Black bars indicate the results of real-time quantitative PCR from three technical replicates. Data are presented as the mean fold change ± standard deviation (SD).

To confirm the structures isolated from serum were exosomes, the morphology, biomarkers, and size distribution were detected. It was revealed that the average size of isolated structures was approximately 74.6 nm in diameter (Fig. 2a left), which was in the range of exosome sizes^11^. The electron micrographs of the exosomes showed rounded structures with a size of approximately 80 nm (Fig. 2a middle). In addition, these vesicles were further confirmed as exosomes by the presence of exosomal marker proteins tumor susceptibility gene 101 protein (TSG101) and CD81 (Fig. 2a right). Thus, the results supported the authenticity of the exosome samples isolated from mice serum.

### Effect of FMT on miRNAs expression in serum exosomes

There were 64 miRNAs differentially expressed in serum exosomes of FMT-treated mice compared with control group (*p* value < 0.05 and absolute fold change >1.5, Supplementary Table S1). Among these miRNAs, two miRNAs were up-regulated and 62 miRNAs were down-regulated (Fig. 2b**)**. As shown in Fig. 2c, the results of hierarchical cluster analysis (HCA) indicated that samples were grouped into two main clusters (FMT-treated mice versus control mice) according to the expressions of the 64 differentially expressed exosomal miRNAs (DEMs). To validate the microarray results, the expressions of five miRNAs were quantified using real-time quantitative PCR, including two up-regulated (miR-23a-3p and miR-215-5p) and three down-regulated (miR-27b-3p, miR-101a-3p, and miR-6394) miRNAs (Fig. 2d). It is demonstrated that the real-time quantitative PCR results are consistent with microarray results.

### Function and pathway analysis of the target genes of DEMs

**Figure 3:**
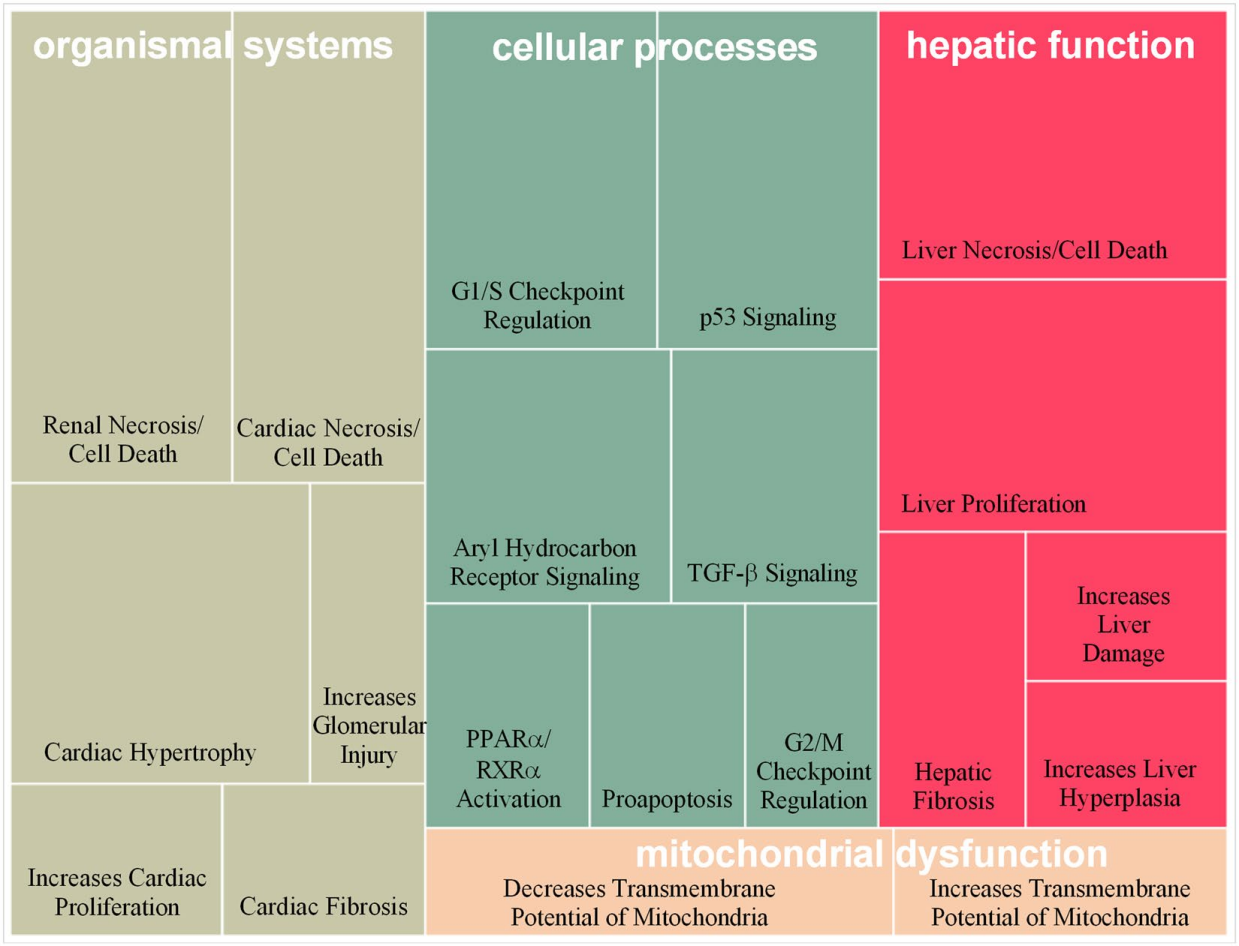
Top 20 toxic lists corresponding to 650 target genes of the 15 DEMs under the treatment of FMT. The size of blocks represented the value of -log(p-value).

Only 15 miRNAs from the 64 DEMs had validated target genes in IPA by target filter analysis, including miR-6349, miR-101a-3p, miR-6394, miR-126a-3p, miR-721, miR-143-3p, miR-497a-5p, miR-93-5p, miR-215-5p, miR-199a-3p, miR-23a-3p, miR-27b-3p, miR-2861, miR-30a-5p, and miR-370-3p. As the results, 650 validated target genes were found and IPA analysis indicated that these target genes were significantly enriched for several cellular functions, such as cell cycle, cellular growth and proliferation, and cellular death and survival (Table 1). The top 20 toxic lists were listed in Fig. 3. Hepatic dysfunction, mitochondrial dysfunction, and pathways related to cell cycle and apoptosis^20–23^ were detected in the toxic lists. Therefore, we speculated that FMT may induce liver cell death through promoting apoptosis by interfering with the normal functions of mitochondria and blocking cell cycle progression.

**Table 1:** Top five cellular functions corresponding to the 650 target genes of the 15 DEMs under the treatment of FMT.

Molecular and Cellular Functions	Number of target genes involved	p-value
Cellular Growth and Proliferation	367	6.77E-53–1.01E-14
Cellular Development	356	1.60E-52–1.17E-14
Cellular Death and Survival	338	7.48E-51–1.86E-14
Cell Cycle	189	1.23E-46–1.44E-14
Cellular Movement	243	3.26E-42–7.33E-15

### Prediction of biological function based on the intersection of exosomal miRNAs targets and the differentially expressed mRNAs (DEGs) in the liver

Our previously published study identified 1,723 DEGs in the FMT-exposed mouse liver compared with control group with the cutoffs of absolute fold change >2 and *p* value < 0.05^10^. These liver DEGs were adopted in this study to understand the hepatic influence of the 64 serum exosomal DEMs. An intersection between liver DEGs and the target mRNAs of serum exosomal DEMs was generated as the miRNA-mRNA intersection dataset. In the dataset, 812 DEGs in liver were corresponded to 29 DEMs in serum exosomes by target filter analysis. The biological and molecular functions of these 812 mRNAs were investigated in IPA, including cellular functions (Table 2), canonical pathways (Table 3), and toxic lists (Table 4). It was worth noting that Nrf2-mediated oxidative stress response was overlapped in toxic lists and canonical pathways. Oxidative stress and apoptosis related pathways such as “Nrf2-mediated oxidative stress response”, “Gadd45 signaling”, and “p53 signaling” were identified in canonical pathways, indicating these pathways may play an important role in FMT-ILI. Based on the relationships between the DEMs and DEGs, there were 24, 9, and 20 miRNAs involved in these three pathways, respectively. Eight miRNAs were induced in common in the three pathways (Table 5). Among them, miR-370-3p was the most down-regulated serum exosomal miRNA by FMT treatment. Therefore, we focused on the functions of this miRNA in the following studies.

**Table 2:** Top five cellular functions of the 812 mRNAs which were the intersection of the target mRNAs of 29 serum exosomal DEMs and the DEGs in liver tissue after FMT exposure.

Molecular and Cellular Functions	Number of genes involved	p-value
Cellular Growth and Proliferation	391	2.89E-32–3.75E-06
Cell Death and Survival	346	7.25E-31–4.99E-06
Cellular Development	366	5.62E-26–3.75E-06
Gene Expression	258	1.54E-24–2.04E-06
Cellular Movement	232	2.18E-21–4.50E-06

**Table 3:** Top five canonical pathways of 812 mRNAs which were the intersection of the target mRNAs of 29 serum exosomal DEMs and the DEGs in liver tissue after FMT exposure.

Canonical pathways	p-value
Nrf2-mediated Oxidative Stress Response	3.22E-7
Activation of IRF by Cytosolic Pattern Recognition Receptors	3.01E-6
Gadd45 Signaling	3.66E-6
p53 Signaling	4.32E-6
Glucocorticoid Receptor Signaling	4.91E-6

**Table 4:** Top five toxic lists of 812 mRNAs which were the intersection of the target mRNAs of 29 serum exosomal DEMs and the DEGs in liver tissue after FMT exposure.

Toxic lists	p-value
Liver Necrosis/Cell Death	3.18E-11
Renal Necrosis/Cell Death	6.32E-9
Acute Renal Failure Panel (Rat)	7.26E-9
Cardiac Hypertrophy	4.69E-8
Nrf2-mediated Oxidative Stress Response	5.83E-8

**Table 5:** The serum exosomal miRNAs lists involved in “Nrf2-mediated Oxidative Stress Response”, “Gadd45 Signaling”, and “p53 Signaling”.

Pathways	miRNAs involved	miRNAs in common
Nrf2-mediated Oxidative Stress Response	miR-497a-5p, miR-7050-5p, miR-93-5p, miR-721, miR-370-3p, miR-30a-5p, miR-7118, miR-6965-5p, miR-6394, miR-6349, miR-149-3p, miR-101a-3p, miR-3473b, miR-143-3p, miR-3154, miR-27b-3p, miR-23a-3p, miR-2861, miR-6769b-5p, miR-215-5p, miR-199a-3p, miR-188-5p, miR-6370, miR-300-5p	miR-370-3p (fold change = −2.64) miR-30a-5p (fold change = −2.14) miR-93-5p (fold change = −2.10) miR-3473b (fold change = −2.02) miR-7118-5p (fold change = −1.94) miR-497a-5p (fold change = −1.94) miR-721 (fold change = −1.83) miR-6349 (fold change = −1.76)
Gadd45 Signaling	miR-721, miR-497a-5p, miR-370-3p, miR-93-5p, miR-6349, miR-30a-5p, miR-3473b, miR-7118-5p, miR-7005-5p	
p53 Signaling	miR-149-3p, miR-101a-3p, miR-143-3p, miR-721, miR-6349, miR-30a-5p, miR-6965-5p, miR-93-5p, miR-3473b, miR-7118-5p, miR-497a-5p, miR-27b-3p, miR-6769b-5p, miR-7050-5p, miR-3154, miR-6394, miR-2861, miR-370-3p, miR-7005-5p, miR-6963-5p	

### Verified experiments–FMT induces S-phase cell cycle arrest in BNL CL.2 cells

As cell line has lots of advantages, such as high stability and reproducibility, there are plenty of studies using cell lines to verify the results from *in vivo* experiments^24,25^. BNL CL.2 is a widely-used immortalized liver cell line derived from the liver of BALB/c mice^26,27^, which were the same mice used in this study. This cell line has hepatic characteristics^26^ and similar responses with primary liver cells^28,29^. Thus, BNL CL.2 cells were chosen to verify the results of pathway analysis.

**Figure 4:**
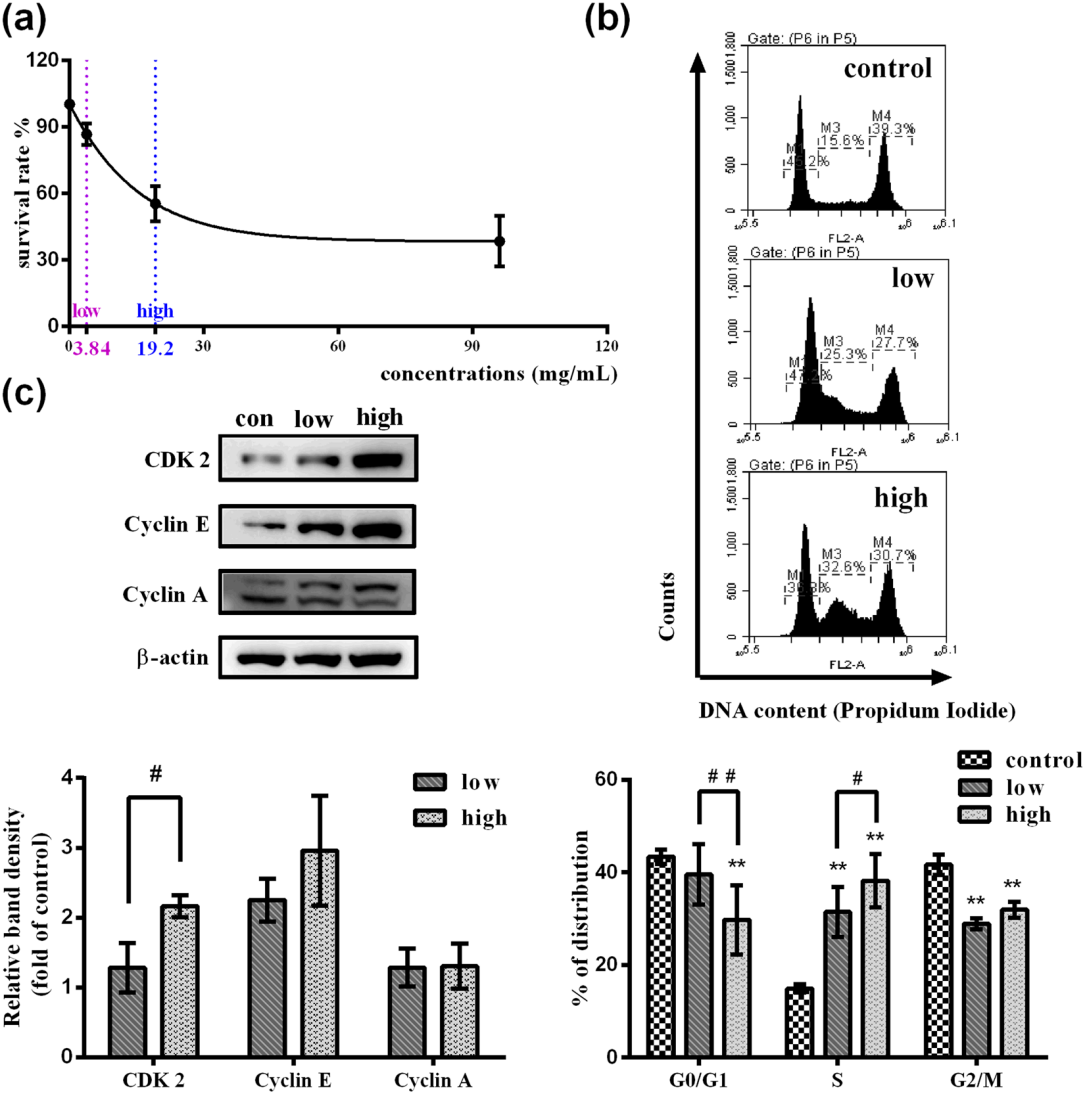
FMT induces S-phase cell cycle arrest in BNL CL.2 cells. (**a**) Survival rates were detected by 3-(4,5-Dimethylthiazol-2-yl)-2,5-diphenyltetrazolium bromide (MTT) assay. The concentrations of 3.84 mg/mL and 19.2 mg/mL were chosen as the low- and high- dose. (**b**) Cell cycle distribution was assessed using flow cytometry. Representative images for cell cycle (upper). The percentages of the cell cycle (below). Two-way ANOVA followed by Tukey Post Test was adopted to determine the differences of cell cycle distributions. (**c**) The expression of S-phase progression-related proteins was determined by western blot. Intensities of bands were normalized to the amount of β-actin. Statistical differences of cellular proteins between diffferent groups were examined by t-test. Data are presented as mean ± SD (n = 3). Full-length western blot images in (**c**) are presented in Supplementary Fig. S2. ^*^p < 0.05, ^**^p < 0.01, versus the control. ^#^*p* < 0.05, ^##^*p* < 0.01, versus the other concentration.

In agreement with results of pathway analysis, we found that FMT perturbed cell cycle *in vitro*. As shown in Fig. 4b, a dose-dependent increase in the proportion of cells in S phase was detected by FMT treatment, accompanied by a concordant decrease in the proportion of cells in G0/G1 and G2/M phases. The increase number of cells in S phase following FMT treatment is more likely due to S-phase arrest rather than cell proliferation because a dose-dependent cytotoxicity of FMT was detected (Fig. 4a).

Cyclins and cyclin dependent kinases (CDKs) regulate cell cycle progression and arrest^30,31^. To explore the molecular mechanisms of FMT-induced S phase arrest, the expressions of S phase cell cycle regulatory proteins were examined by western blot. Cyclin dependent kinase 2 (CDK2) increased significantly in a dose-dependent manner upon treatment with FMT for 48 h, whereas the increase of Cyclin E did not have the obvious dose-effect relationship (Fig. 4c). However, the levels of Cyclin A were not affected (Fig. 4c).

### Verified experiments–FMT causes apoptosis in BNL CL.2 cells

**Figure 5:**
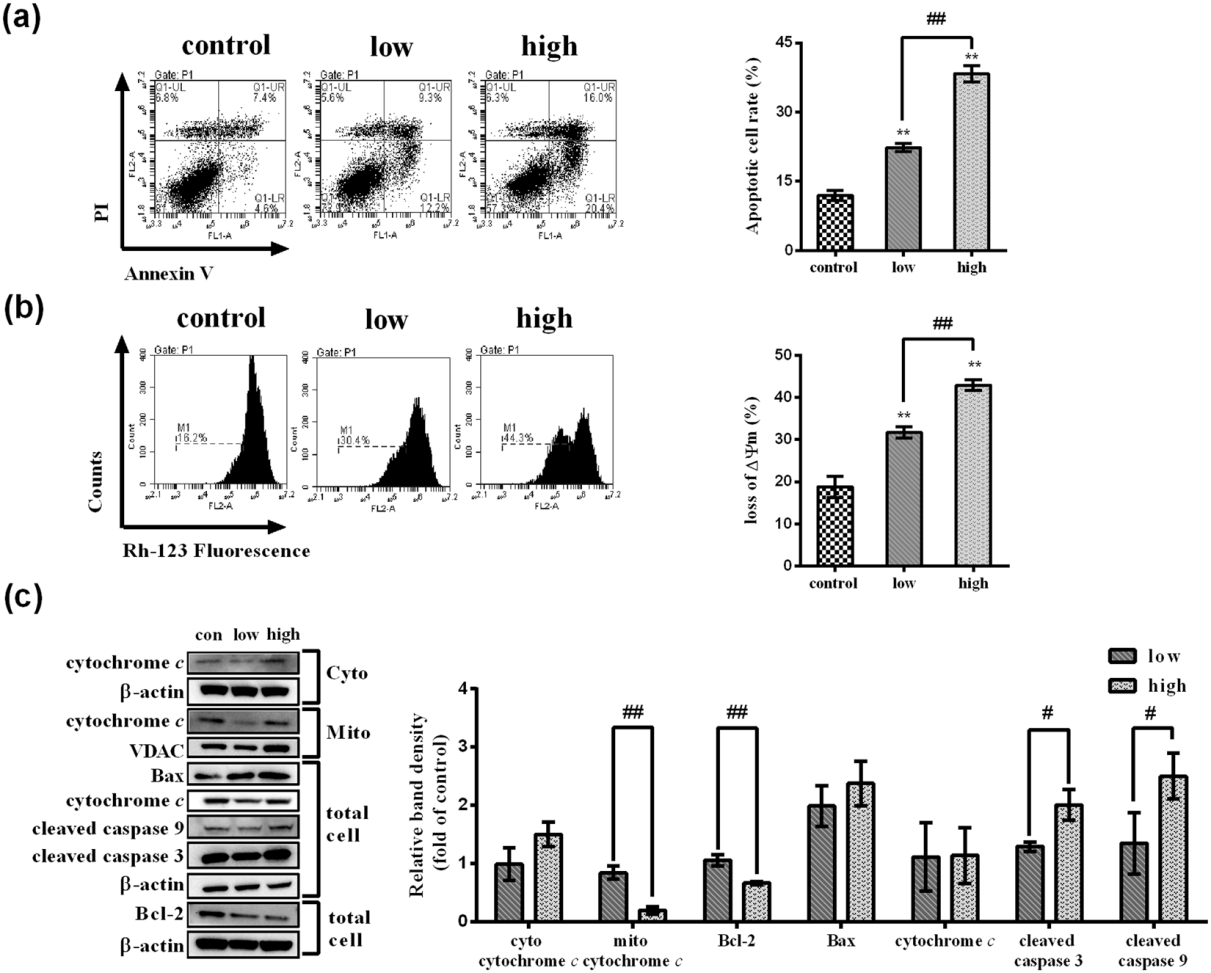
Effects of FMT on apoptosis and mitochondrial function in BNL CL.2 cells. (**a**) The rates of apoptosis were assessed using the Annexin V-FITC/PI dual-labeling techniques. Representative images (left). The apoptotic rates (right). One-way ANOVA followed by Tukey Post Test was adopted to examine the statistical differences. (**b**) The loss of ΔΨ m was detected using Rh-123. One-way ANOVA followed by Tukey Post Test was adopted to examine the statistical differences. (**c**) The expressions of mitochondrial dysfunction-related proteins in mitochondrial fractions, cytosolic fractions, and the whole cells were measured. β-actin was used for normalization and verification of cytosolic fractions and the whole cells loading. Meanwhile, the protein of voltage-dependent anion channel (VDAC) was used for normalization and verification of mitochondrial fractions loading. Statistical differences between different groups were examined by t-test. Data are shown as mean ± SD (n = 3). Full-length western blot images in (**c**) are presented in Supplementary Figs S3 and 4. ^*^p < 0.05, ^**^p < 0.01, versus the control. ^#^*p* < 0.05, ^##^*p* < 0.01, versus the other concentration.

Our pathway analysis suggested that cell apoptosis may be involved in FMT-ILI, thus the apoptosis of BNL CL.2 cells after exposure to FMT was detected by Annexin V-fluorescein isothiocyanate (FITC)/propidium iodide (PI) assay. As shown in Fig. 5a, the proportions of apoptotic cells were increased in a dose-dependent manner after FMT treatments (*p* < 0.01).

### Verified experiments–FMT leads to mitochondria damage in BNL CL.2 cells

A reduced mitochondrial transmembrane potential (∆Ψm) is associated with mitochondrial dysfunction linked to apoptosis^32^. As mitochondrial dysfunction was also enriched in the pathway analysis, the alterations of fluorescent intensity of rhodamine-123 (Rh-123) were examined to describe the ΔΨm variation with FMT treatment. As shown in Fig. 5b, BNL CL.2 cells exhibited a remarkable reduction in Rh-123 fluorescence (*p* < 0.01) after incubated with FMT, suggesting the loss of ΔΨm across the membrane. In addition, a dose-dependent ΔΨm loss was detected.

The release of cytochrome *c* from mitochondria into the cytosol is one of the major apoptosis pathways. Cytosolic cytochrome *c* induces cysteine-aspartic acid protease 9 (caspase 9)-dependent activation of caspase 3. As shown in Fig. 5c, the levels of cytosolic cytochrome *c* were increased and the expression of cytochrome *c* in mitochondrial was decreased after FMT administration. It suggested a subcellular translocation of cytochrome *c* from mitochondrial into the cytosol. Moreover, FMT exposure increased the levels of cleaved caspase 9 and cleaved caspase 3 in a dose-dependent manner (Fig. 5c). There was only one blot of the cleaved caspase 9 and cleaved caspase 3. This phenomenon was also observed by previous studies^33,34^.

Taken together, these results indicate that FMT induces apoptosis via the caspase- and mitochondrial-dependent pathway in BNL CL.2 cells.

### Verified experiments–FMT affects the pathways indicated in IPA based on biological function analysis of the intersection of exosomal miRNAs targets and DEGs in the liver

**Figure 6:**
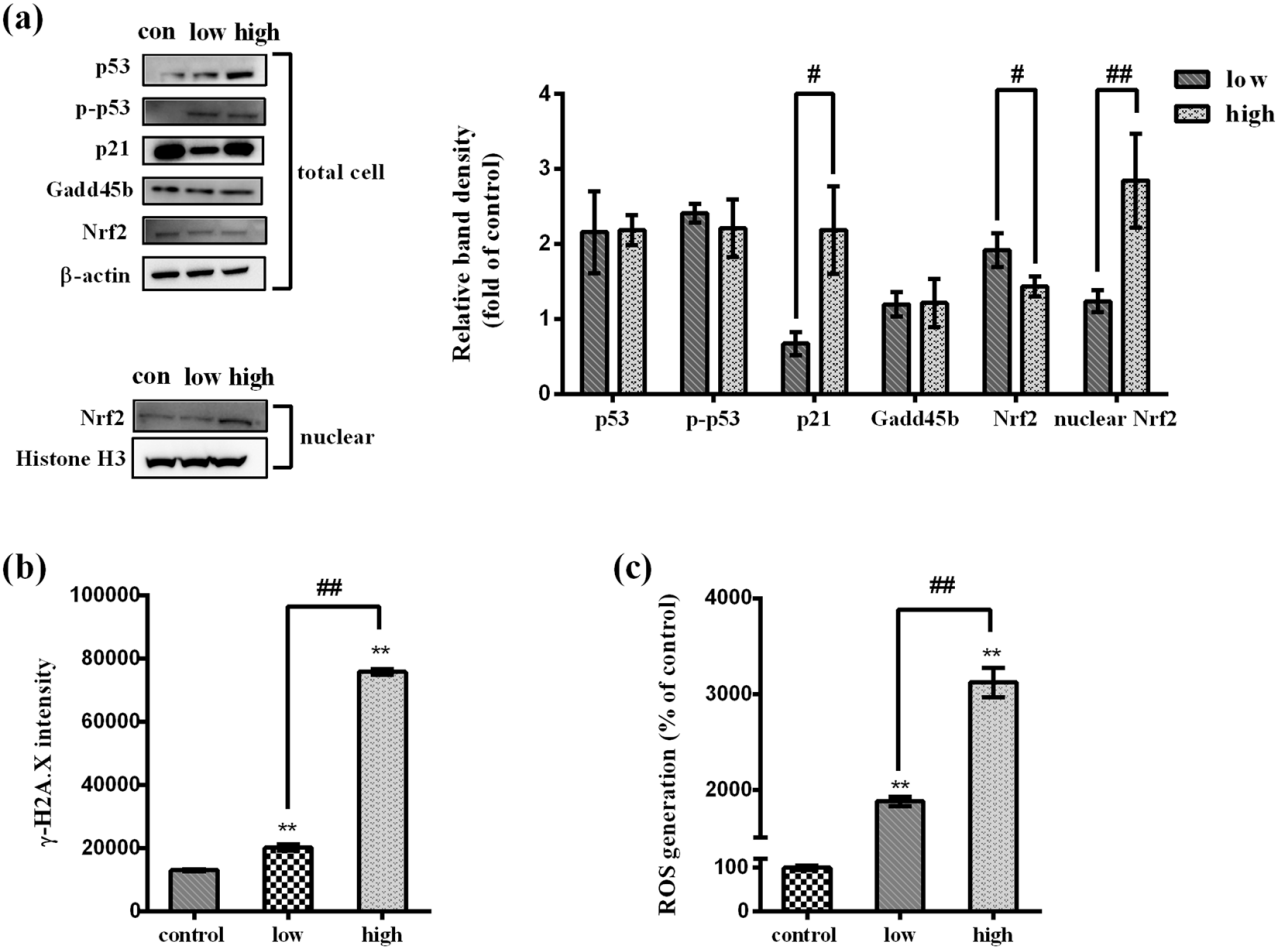
Effect of FMT on protein levels, DNA damage, and ROS generation in BNL CL.2 cells. (**a**) The protein expressions in the whole cells and nuclear fractions were detected. β-actin was used for normalization and verification of the whole cells loading. Meanwhile, histone H3 was used for normalization and verification of nuclear fractions loading. Statistical differences of cellular proteins between different groups were examined by t-test. Full-length western blot images are presented in Supplementary Fig. S5-6. (**b**) The fluorescence of the cells stained with FITC-γ-H2A.X was analyzed by flow cytometry. One-way ANOVA followed by Tukey Post Test was adopted to examine the statistical differences. (**c**) The fluorescence of the cells stained with DCFH-DA was analyzed using flow cytometry. One-way ANOVA followed by Tukey Post Test was adopted to examine the statistical differences. Data are presented as mean ± SD of three independent experiments. ^*^p < 0.05, ^**^p < 0.01, versus the control. ^#^*p* < 0.05, ^##^*p* < 0.01, versus the other concentration.

The IPA analysis results suggested three important pathways involved in FMT-ILI (Table 3 and Table 4), including Gadd45 signaling, p53 signaling, and Nrf2-mediated oxidative stress response. In order to investigate whether these pathways were disturbed by FMT, the key proteins in these pathways were examined by western blot after BNL CL.2 cells exposed to low- and high-dose FMT for 48 h. As shown in Fig. 6a, the levels of Gadd45b were not affected by FMT administration, but FMT increased the levels of phosphorylated p53 (p-p53) and p53. The protein levels of p21, a key protein in p53 pathway, changed conversely in low- and high-dose group with significant differences. Furthermore, treatment with FMT significantly increased the expression of Nrf2 either in the whole cells or in the nucleus. The fraction of Nrf2 in the nucleus was increased in a dose dependent manner while the Nrf2 proteins in the whole cells were not. These results indicated that FMT treatment upregulated Nrf2 expression and promoted Nrf2 translocation into cell nucleus. Taken together, the Nrf2 mediated signaling pathway and p53 signaling pathway are activated in BNL CL.2 cells when stimulated by FMT.

It has been reported that S-phase arrest may be accompanied by DNA damage^35^ which can result from ROS accumulation. Thus, DNA damage and ROS generation were examined using flow cytometry. The presence of DNA strand breaks was confirmed by measuring phosphorylate histone H2A.X (γ-H2A.X), a hallmark of DNA double-strand breakage in cells^36^. Treatment of BNL CL.2 cells with FMT for 48 h displayed a dose-dependent upregulation of γ-H2A.X (Fig. 6b), pointing out the significant DNA damage. ROS levels were evaluated using 2′-7′-dichlorodihydrofluorescein diacetate (DCFH-DA) dyes. The results showed that FMT induced dose-dependent increases in ROS levels (Fig. 6c). The intracellular ROS generation was 19 times higher in low-dose FMT-treated group and 31 times higher in high-dose FMT-treated group than that in the control group.

## Discussion

In recent years, abundant studies have provided strong evidence that exosomal miRNAs are novel biomarkers and therapy targets for various diseases. miRNAs in circulating exosomes are protected from degradation even with the high ribonuclease activity in blood^37^. This remarkable stability of circulating exosomal miRNAs raises the possibility of using them as biomarkers. Clinical studies have proved that circulating miRNAs in exosomes are potential biomarkers for diagnosis, identification of subtype, evaluation of severity, and predicting prognosis of diseases^38–40^. Furthermore, circulating exosomal miRNAs are usually regarded as cell-to-cell communicators and cell signaling triggers in spite of the distance between cells^41,42^. Hence, monitoring the miRNAs in circulating exosomes is useful to understand the mechanisms and the progresses of diseases^42–44^.

The objective of this study was to further investigate the mechanisms of FMT-ILI by analysis of circulating exosomal miRNAs. There are 64 miRNAs in mouse serum exosomes that are found significantly up- or down-regulated by the administration of FMT. Pathway analysis suggested that hepatic dysfunction of FMT-treated mice may be due to apoptosis, mitochondrial dysfunction, and cell cycle arrest (Table 1 and Fig. 3). As hepatoxicity is the main toxicity effect of FMT, the profiles of DEGs in liver were integrated to explore the impact of the 64 serum exosomal miRNAs. As the results, oxidative stress and apoptosis related pathways were significantly enriched, including Nrf2-mediated oxidative stress response, Gadd45 signaling, and p53 signaling (Table 3 and Table 4).

Corresponding to the results of pathway analysis, FMT-induced oxidative stress was detected as ROS production increased in a dose-dependent manner in BNL CL.2 cells (Fig. 6c). ROS generation is essential in the initiation and progression of liver pathologies including those caused by alcohol, hepatic viruses, and drugs^45–47^. Under normal physiologic conditions, cells maintain redox homeostasis through generation and elimination of ROS. On one hand, in response to ROS, several defensive signaling pathways are activated for cell survival. The well-studied Nrf2 signaling pathway is the major survival mechanism that provides defensive responses against oxidative stress and serves as ROS scavengers^48^. Normally, cells maintain the Nrf2 protein at a low level. When excessive ROS is generated, Nrf2 is increased^49^ and translocated into nucleus to trans-activate its target genes^50^. In our study, a marked accumulation of total Nrf2 protein and the nuclear translocation of Nrf2 were observed (Fig. 6a), indicating the Nrf2 upregulation and translocation. On the other hand, superabundant ROS triggers DNA damage and then initiates a series of cascade reactions^51^, such as cell apoptosis or cell cycle arrest^52^. The early events in apoptosis and cell cycle arrest are highly conserved. One of the most important early events is the activation of tumor suppressor protein p53^53^. p53 is also called as guardian of the genome because it initiates expression of those genes that govern cell cycle arrest, DNA damage repair, and apoptosis. In this study, we demonstrated that FMT augmented the formation of DNA damage marker (γ-H2A.X) and upregulated the expressions of total p53 and p-p53 *in vitro* (Fig. 6). The p-p53 signals nuclear DNA damage to mitochondria^53^. Once at the mitochondrial outer membrane, p-p53 appears to enhance the action of proapoptotic proteins such as Bcl2 Associated X (Bax) and suppress the action of antiapoptotic proteins such as Bcl-2^54^. The decreased Bcl-2 also raises the expression of Bax^55^. This increased Bax leads to the drop of mitochondrial membrane potential, the release of cytochrome *c*, and the activation of caspase 9 and caspase 3 sequentially, which were observed in this study (Fig. 5). Therefore, cell apoptosis occurs (Fig. 5). In addition, the p-p53 activates p21 transcriptionally, accompanied with the disturbation of Cyclin and CDK complexes^56^. Then cell cycle arrest occurs. In our results, S-phase block in cell cycle was confirmed by flow cytometry (Fig. 4b). FMT treatment promoted the expression of p21 in high-dose concentration group while suppressed it in low-dose group (Fig. 6a). As shown in Fig. 4c, FMT markedly increased the levels of Cyclin E and CDK2, which was not coincided with the effect of enhancive p21. We suggest that the FMT increases the level of CDK2 and Cyclin E in some other ways. Based on published literatures, the level of CDK2 increases in S phase when the cell is exposed to DNA damage^30^, and Cyclin E accumulates when S phase arrest happens^31^. Activation of CDK2 is also responsible for cell apoptosis^57–59^.

The results mentioned above collectively suggested the activation of Nrf2-mediated oxidative stress response and p53 signaling pathway after FMT treatment. Both of these two pathways could be regulated by 8 miRNAs, and miR-370-3p was the most dramatically changed miRNAs (Table 5). miR-370-3p is a liver-abundant miRNA^60^ that plays a pivotal role in the maintenance of liver homeostasis^61,62^. Cyclin E and p21 were the predicted target genes of miR-370-5p in IPA dataset. Actually, the interactions between miR-370-5p and these two target genes have been already verified by ChIP assay or luciferase reporter assay in previous study^63,64^. In this study, the administration of FMT decreases the level of circulating exosomal miR-370-5p. Consequently, the inhibition of target p21 and Cyclin E in liver cells is abated. The liver injury is further intensified by the increased p21 and Cyclin E. A recent study also showed that restoration of reduced miR-370-3p had beneficial effects on the treatment of liver diseases^60^, which was consistent with our results. Interestingly, we notice that miR-370-3p in liver tissue did not show significant differences between the FMT-treated and control groups in our previous study^10^. This could be due to the characteristics of liver. Liver is composed of various cells, such as sinusoidal endothelial cells, hepatocytes, hepatic stellate cells, and kuffer cells. Although some specific cells probably internalize the circulating exosomes with lower miR-370-3p, the alterations of miR-370-3p are neutralized to changelessness when the whole liver is used as sample source.

**Figure 7:**
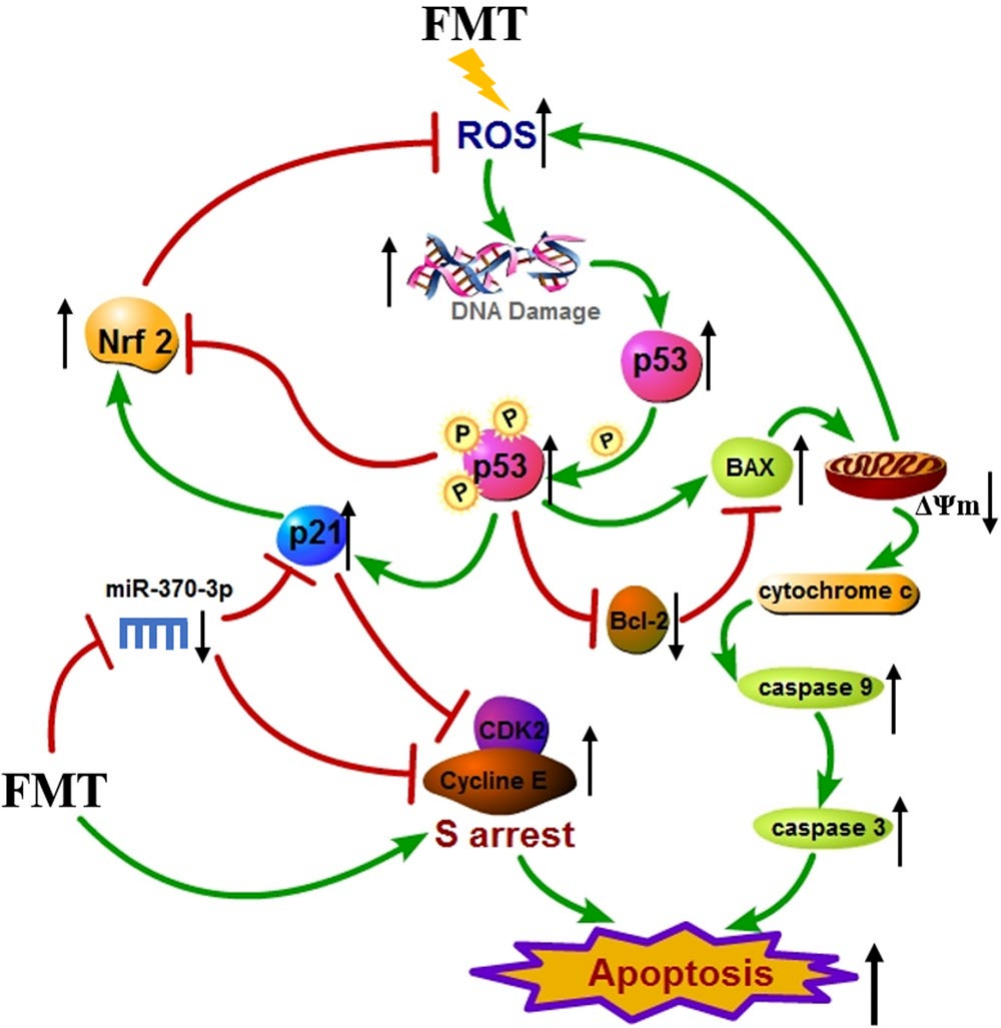
Ingenuity network diagram depicting interactions between the components of the p53 and Nrf2-mediated oxidative stress signaling pathways. For visualization, network was mainly restricted to molecules showing regulation during treatment. Lines indicate known interactions between molecules (red lines depict stimulation and green lines depict suppression). Black up or down arrows represent the behaviors of specific molecules.

Taken together, we present a schematic view of the pathways inside cells and pinpoint where miR-370-3p could perform its actions in Fig. 7. FMT-induced ROS generation causes DNA damage, which leads apoptosis through p53-dependent mitochondrial damage and S-phase arrest. To counteract this impairment, Nrf2-mediated oxidative stress response is activated to protect cells/liver from FMT-induced oxidative damage. Moreover, the reduced miR-370-3p in circulating exosomes may be the momentous event in aggravating cytotoxic effect of FMT by targeting p21 and Cyclin E.

In conclusion, this study demonstrated that the serum exosomal miRNAs profiles further provide insights into FMT-ILI, suggesting miRNAs profiles in circulating exosomes could be a useful tool to understand the mechanisms of TCM-ILI as well as DILI.

## Materials and Methods

### Animals treatment and serum collection

The extraction of FMT and animal treatment are described in details in a previous study^10^. Briefly, 83 mL water extract was obtained from 1 kg dried FMT (Zhejiang Chinese Medical University Medicine Plant, China) through hot water extraction and ethanol precipitation. As toosendanin is one of the major hepatotoxic compounds in FMT, its content in the water extract of FMT was determined by HPLC. The results showed that the content of toosendanin was 0.51 mg/g in the water extract of FMT. Male BALB/c mice were randomly divided into the vehicle control group and the FMT group according to the body weight (n = 15). Mice were intragastrically administrated with FMT (240 g/kg, an equivalent amount of the crude drug) or the solvent (1% sodium carboxymethyl cellulose) once a day for three consecutive days. Blood samples were acquired from inferior vena cava after 6 h of the last administration. Serum was collected for exosomes isolation and biochemical determination. The animal experiments were approved by the Animal Care and Use Committee of Zhejiang University School of Medicine and conducted in accordance with the guiding principles covered in the Use of Animals in Toxicology.

### Serum exosomes isolation

To obtain enough exosomes, the serum samples more than 300 μL were picked out for the further experiments. There were 12 eligible serum samples in each group. Three hundred microliter serum from each sample was collected. Then serum samples of three mice from the same group were mixed together. Thus, four mixed samples in each group were used for exosome isolation. Serum exosomes were isolated using ExoQuick^TM^ Precipitation Solution (System Biosciences, USA) according to the manufacturer’s recommendations. In brief, 227 µL ExoQuick^TM^ Precipitation Solution was added to 900 µL serum mixture and then refrigerated for 30 min. At the end of the incubation time, the samples were centrifuged at 1500 g for 30 min. Then the pellets of exosome-rich fraction underwent a second round of centrifugation at 1500 g for 5 min and dissolved in 100 µL phosphate buffered saline (PBS) for further analysis.

### Exosomes characterization

The morphology of exosomes was examined by a JEM-1230 transmission electron microscopy (JEOL, Japan). A 20 μL drop of the exosomes suspension was loaded onto formvar-carbon-coated electron microscopy copper grids at room temperature. Excess fluid was drawn off with a piece of filter paper. The sample was then negative-stained with a 20 μL drop of 1% uranyl acetate for 1 min and allowed to dry before viewed in transmission electron microscope at 80 kiloelectron volts.

The biomarkers of exosomes were detected by western blot. The samples were resuspended in 4 × loading buffer, separated by 10% sodium dodecyl sulfate polyacrylamide gel electrophoresis (SDS-PAGE) and developed using anti-TSG101 (Abcam, UK) and anti-CD81 (Abcam, UK) antibodies followed by goat anti-rabbit IgG coupled to horseradish peroxidase (Beyotime Biotechnology, China). Peroxidase activity was observed using a charge-coupled device camera (Bio-Rad, USA).

The size determination of isolated serum exosomes was performed using dynamic light scattering by a Zetasizer Nano S90 (Malvern, UK) in accordance with the manufacturer’s instructions.

### Exosomal miRNAs isolation and miRNAs expression analysis

mirVana^TM^ PARIS^TM^ Kit (Ambion, USA) was used to extract the total RNA (including miRNAs) from serum exosomes according to the manufacturer’s instructions. The expression levels of miRNAs were detected with Agilent mouse miRNA (8*60 K) V21.0 array. Microaray raw data were normalized by Gene Spring GX 12 (Agilent technologies, USA) and then analyzed by *t* test. The miRNAs with an absolute fold change greater than 1.5-fold and *p* value less than 0.05 between the control and FMT groups were defined as the DEMs. The HCA within ArrayTrack^®^ 3.5.0 was performed based on the expression levels of DEMs for visualizing the cluster of FMT-treated and the control samples.

### Real-time quantitative PCR

Real-time quantitative PCR was performed on an Eppendorf Mastercycler ep realplex^4^ to validate the results of microarray analysis as described previously^10^ with slight modifications. miScript II RT Kit (Qiagen, Germany) was used for miRNAs reverse transcription. miScript SYBR Green PCR Kit (Qiagen, Germany) and individual specific primers (XY Biotechnology, China) were used for miRNAs expression detection according to the manufacturer’s recommendations. The primer sequences were shown in Table 6. miR-20b-5p and miR-15b-5p were chosen as the internal references for normalization due to their stable expressions in every sample. Geometric mean of Ct_miR-20b-5p_ and Ct_miR-15b-5p_ was used as a normalizer for miRNAs expression. The fold change was generated by 2^−ΔΔCt^ method.

**Table 6:** The sequences of miRNA primers.

miRNAs	Primer sequence (5′-3′)
miR-23a-3p	ATCACATTGCCAGGGATTTCC
miR-215-5p	ATGACCTATGATTTGACAGAC
miR-27b-3p	TTCACAGTGGCTAAGTTCTGC
miR-101a-3p	TACAGTACTGTGATAACTGAA
miR-6394	TCCCTGAGTGGGGCCAGGTCT
miR-20b-5p	CAAAGTGCTCATAGTGCAGGTAG
miR-15b-5p	TAGCAGCACATCATGGTTTACA

### Biological pathways analysis

DEMs in FMT treated group were uploaded into IPA (Ingenuity Systems, USA) for further analyses. The first step was to detect the target mRNAs of the DEMs by “Target Filter” in IPA, which identified experimentally validated miRNA-mRNA interactions from TarBase, miRecords, and the peer-reviewed biomedical literature. The second step was to explore the functions of these target mRNAs by “tox analysis” in IPA, including toxic lists, canonical pathways, and diseases and disorders.

Considering liver is the main target organ of FMT-induced toxicity demonstrated by our previous study^10^, we are interested in the changes in liver tissue accompanied by the alterations in serum exosomes. Thus, we built a target genes dataset including the experimentally validated and highly and moderately conserved predicted target mRNAs of DEMs in serum exosomes and then linked the target genes dataset with the DEGs in liver tissue which were reported previously^10^. The IPA analysis was performed based on the intersection of these two parts.

### Cell culture

The murine embryonic liver cell line BNL CL.2 was purchased from the Cell Bank of Shanghai Institutes for Biological Sciences, Chinese Academy of Sciences. Cells were cultured in complete Dulbecco’s Modified Eagle Medium (DMEM, Gibco, USA) culture medium supplemented with 10% fetal bovine serum (Gibco, USA) and 1% penicillin/streptomycin (Gibco, USA). The cells were kept at 37 °C in a humidified atmosphere incubator containing 5% carbon dioxide.

### *In vitro* cell viability assay

The *in vitro* growth-inhibitory effect of FMT was measured by MTT assay (Sigma, USA). BNL CL.2 cells were seeded in 96-well flat bottom plates at a density of 5 × 10^3^ cells per well. After 24 h, cells were treated with different concentrations of FMT in 100 µL of fresh culture medium for 48 h. The non-cell wells containing PBS were used as blank and the cell wells containing fresh culture medium were regarded as control. Then the medium was replaced with 100 µL of fresh culture medium containing MTT (0.5 mg/mL). After a 4-h incubation at 37 °C, MTT-containing medium was removed and 100 µL of dimethyl sulfoxide were added to each well to solubilize the purple formazan crystals. The optical density (OD) was read at 580 nm using Infinite M1000 Pro (TECAN, Germany). The treatment groups were compared with the control group, and the survival rate was calculated using the formula, survival rate (%) = (OD_FMT_ − OD_blank_)/(OD_control_ − OD_blank_) × 100%.

### Cell cycle analysis

BNL CL.2 cells (1.5 × 10^5^ cells per well) were cultured in 6 well plates and allowed to adhere overnight. After incubation for 24 h, cell cycle synchronization was carried out by serum starvation for 24 h at 37 °C and 5% CO_2_. Then the cells were treated with FMT for 48 h at the doses of 3.84 mg/mL (low-dose) and 19.2 mg/mL (high-dose), which were presented as an equivalent amount of the crude drug in culture medium. Complete DMEM culture medium treated cells were served as control. Subsequently, cells were harvested and fixed in 70% chilled alcohol overnight, washed twice with PBS, digested with DNase-free RNase and stained with PI for 30 min at 37 °C in dark according to the manufacturer’s protocol (Cell Cycle and Apoptosis Analysis Kit; Beyotime Biotechnology, China). The cell cycles were analyzed using Accuri^™^ C6 flow cytometer (BD, USA) to collect 20,000 events.

### Cell apoptosis assay

For the quantitative analysis of apoptosis, the BNL CL.2 cells were detected using the Annexin V-FITC/PI Apoptosis Detection kit (BD Pharmingen, USA) with double staining according to the manufacturer’s instructions. Briefly, BNL CL.2 cells were seeded at a density of 1.5 × 10^5^ cells per well in 6-well plates and treated with 3.84 mg/mL (low-dose) and 19.2 mg/mL (high-dose) FMT for 48 h. Then the cells were collected and resuspended in 100 µL binding buffer. Cells were analyzed by Accuri^™^ C6 flow cytometer (BD, USA) after incubation with 5 µL of FITC-conjugated Annexin V and 5 µL of PI for 15 min at room temperature in dark. Cells staining positive for Annexin V and negative for PI were identified as early apoptotic cells. Cells stained positive for both Annexin V and PI were regarded as late apoptotic cells. The sum of the early apoptotic rate and late apoptotic rate was used to describe apoptotic rate.

### Measurement of the mitochondrial transmembrane potential

Variations of ∆Ψm as a result of mitochondrial perturbation induced were measured after staining with Rh-123 (Sigma, USA). BNL CL.2 cells were maintained at a density of 1.5 × 10^5^ cells per well in 6 well plates for 24 h and treated with 3.84 mg/mL (low-dose) and 19.2 mg/mL (high-dose) FMT for 48 h. After collected and washed twice with PBS, the cells were incubated with Rh-123 (5 μg/mL) in dark for 30 min at 37 °C. Then the cells were washed twice with DMEM and analyzed by Accuri^™^ C6 flow cytometer.

### Intracellular γ-H2A.X determination

H2A.X phosphorylation was analyzed by flow cytometry analysis. BNL CL.2 cells were maintained at a density of 1.5 × 10^5^ cells/well in 6-well plates for 24 h and treated with 3.84 mg/mL (low-dose) and 19.2 mg/mL (high-dose) FMT for 48 h. The cells were collected and washed twice with PBS followed by fixation in 4% paraformaldehyde for 15 min. Then the cells were stained in 0.3% Triton X-100 for 15 min followed by staining with 5 μL FITC anti-Histone H2A.X phosphorylation at serine 139 antibody (Biolegend, USA) in darkness for 30 min at room temperature. The samples were then analyzed on Accuri^™^ C6 flow cytometer.

### Intracellular reactive oxygen species (ROS) determination

Changes in intracellular ROS levels were determined by measuring the oxidative conversion of DCFH-DA to fluorescent dichlorofluorescein by flow cytometry analysis. BNL CL.2 cells were maintained at a density of 1.5 × 10^5^ cells/well in 6-well plates for 24 h and treated with 3.84 mg/mL (low-dose) and 19.2 mg/mL (high-dose) FMT for 48 h. The cells were washed twice with PBS and incubated with DCFH-DA (Beyotime Biotechnology, China) at 37 °C in dark for 20 min. Then DCF fluorescence distribution of 10,000 cells was detected by Accuri^™^ C6 flow cytometer.

### Western blot analysis

Total proteins of BNL CL.2 cells were lysed in Western and IP lysis buffer containing protease inhibitor cocktail (Roche Diagnostics, Germany) after treatment with FMT for 48 h. NE-PER nuclear and cytoplasmatic extraction reagents (Thermo Fisher Scientific, USA) were used to isolate nuclear fractions. Isolation of mitochondrial and cytosolic proteins was performed using the Mitochondria/cytosol Fractionation Kit (Beyotime Biotechnology, China). The protein concentration was determined using a Bicinchoninic Acid Protein Assay Kit (Thermo Fisher Scientific, USA). Reduced 10% SDS-PAGE was performed and samples were transferred to polyvinyliden difluoride membrane. After blocking with TBST (Tris-buffered saline, 0.1% Tween 20) containing 5% fat-free milk, the membranes were incubated with the primary antibody against p21 (Boster Biological Technology, China), CDK2 (Boster Biological Technology, China), Cyclin A (Absin, China), Cyclin E (Absin, China), Gadd45b (Absin, China), p-p53 (Cell Signaling, USA), p53 (Cell Signaling, USA), Nrf2 (Cell Signaling, USA), caspase 3 (Cell Signaling, USA), caspase 9 (Cell Signaling, USA), Bax (Cell Signaling, USA), Bcl-2 (Cell Signaling, USA), cytochrome *c* (Cell Signaling, USA), VDAC (Cell Signaling, USA), histone H3 (Cell Signaling, USA), and β-actin (Cell Signaling, USA). The blots were then reacted with HRP-conjugated antibody for 1 h at room temperature and detected with the enhanced chemiluminescent substrate reagent (Thermo Fisher Scientific, USA). Protein bands were visualized and digitized using a charge-coupled device camera (Bio-Rad, USA).

### Statistical analysis

Data are expressed as mean ± SD of three independent experiments. Statistical differences were examined by t-test, two-way ANOVA followed by Tukey Post Test, or one-way ANOVA followed by Tukey Post Test. A *p* < 0.05 was considered to be significant.

### Data availability

All data generated or analyzed during this study are included in this published article.

## Electronic supplementary material

Supplementary Material
