# Supplementary material for: Circulating exosomal microRNAs reveal the mechanism of Fructus Meliae Toosendan-induced liver injury in mice
Source: Sci Rep. 2018 Feb 12;8:2832. doi: 10.1038/s41598-018-21113-6 (PMC5809479; doi:10.1038/s41598-018-21113-6)
Supplement: Supplementary file 1 — Supplementary Material [file 41598_2018_21113_MOESM1_ESM.pdf]

Supplementary Materials

Circulating exosomal microRNAs reveal the mechanism of Fructus Meliae Toosendan-induced liver injury in mice

Jie Zheng[1], Lingqi Yu[1], Wen Chen[1], Xiaoyan Lu[1]*, Xiaohui Fan[1]*

[1]Pharmaceutical Informatics Institute, College of Pharmaceutical Sciences, Zhejiang University, Hangzhou, China.

* Correspondence: **Assoc Prof. Xiaoyan Lu (luxy@zju.edu.cn) and Prof. Xiaohui Fan (fanxh@zju.edu.cn)**

Supplementary Table S1: Lists of affected miRNAs in mouse serum exosomes by FMT treatment

miRNA	p-value	Fold Change
miR-23a-3p	0.032	2.310
miR-215-5p	0.033	2.167
miR-6963-5p	0.026	-1.511
miR-1892	0.040	-1.522
miR-5112	0.015	-1.523
miR-27b-3p	0.032	-1.547
miR-7088-5p	0.001	-1.562
miR-8119	0.049	-1.587
miR-149-3p	0.035	-1.588
miR-101c	0.045	-1.623
miR-30e-5p	0.038	-1.700
miR-7672-5p	0.025	-1.728
miR-2861	0.014	-1.735
miR-300-5p	0.020	-1.747
miR-6349	0.027	-1.756
miR-6995-5p	0.006	-1.767
miR-3473e	0.037	-1.773
miR-106b-5p	0.028	-1.791
miR-6394	0.035	-1.831
miR-721	0.042	-1.834
miR-6378	0.017	-1.849
miR-199a-3p	0.036	-1.857
miR-5119	0.009	-1.874
miR-101a-3p	0.006	-1.895
miR-7045-5p	0.016	-1.904
miR-6965-5p	0.048	-1.932
miR-7118-5p	0.000	-1.940
miR-6987-5p	0.017	-1.940
miR-497a-5p	0.003	-1.941
miR-7039-5p	0.009	-1.941

miRNA	p-value	Fold Change
miR-8105	0.006	-1.947
miR-6769b-5p	0.029	-1.950
miR-712-5p	0.012	-1.970
miR-710	0.031	-1.982
miR-3473b	0.008	-2.018
miR-5100	0.025	-2.032
miR-7050-5p	0.042	-2.048
miR-6908-5p	0.018	-2.083
miR-93-5p	0.016	-2.105
miR-30a-5p	0.017	-2.140
miR-126a-3p	0.030	-2.157
miR-143-3p	0.023	-2.159
miR-6366	0.011	-2.168
miR-5131	0.013	-2.233
miR-1904	0.038	-2.378
miR-188-5p	0.035	-2.379
miR-6969-5p	0.049	-2.399
miR-3081-5p	0.023	-2.442
miR-6368	0.017	-2.451
miR-452-5p	0.000	-2.452
miR-6392-3p	0.015	-2.470
miR-7241-3p	0.011	-2.479
miR-6370	0.025	-2.520
miR-7005-5p	0.035	-2.576
miR-2137	0.035	-2.602
miR-125a-3p	0.017	-2.622
miR-6898-5p	0.020	-2.637
miR-370-3p	0.018	-2.640
miR-3154	0.020	-3.328
miR-680	0.015	-3.466
miR-5622-3p	0.014	-3.845

miRNA	p-value	Fold Change
miR-7036a-5p	0.010	-4.020
miR-6944-5p	0.013	-4.036
miR-208a-5p	0.001	-4.059

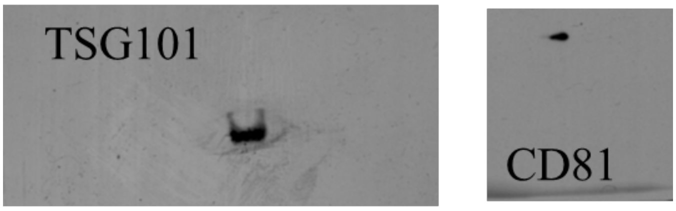

Figure S1. Full-length blots used to generate Figure 2a.

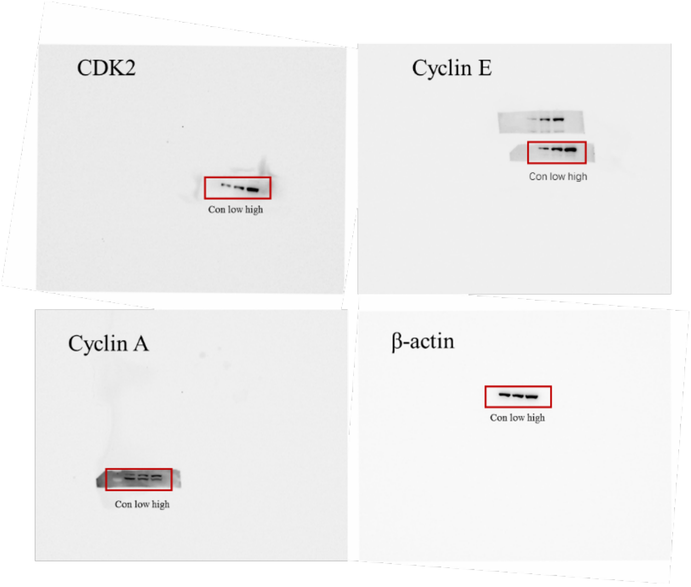

Figure S2. Full-length blots used to generate Figure 4c.

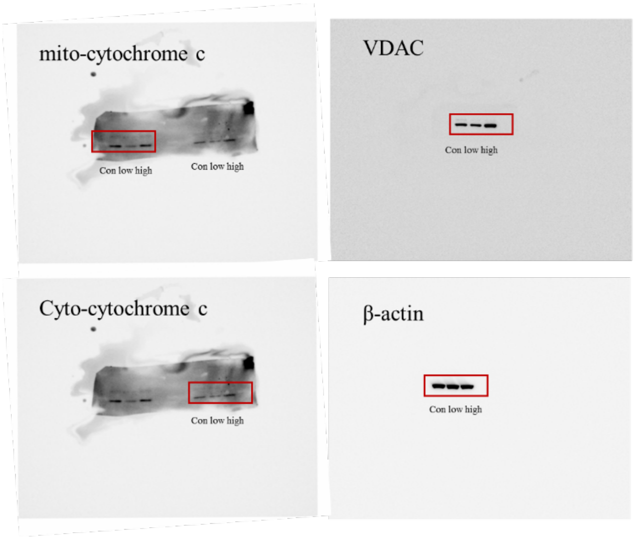

Figure S3. Full-length blots used to generate Figure 5c-cyto and mito proteins.

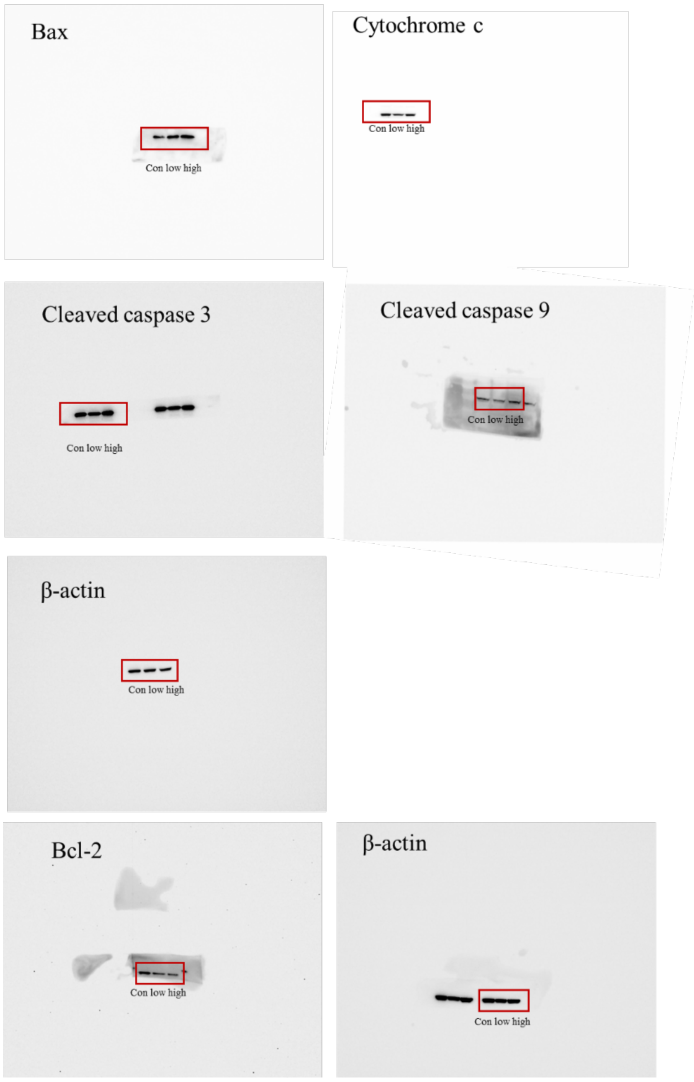

Figure S4. Full-length blots used to generate Figure 5c-total cell.

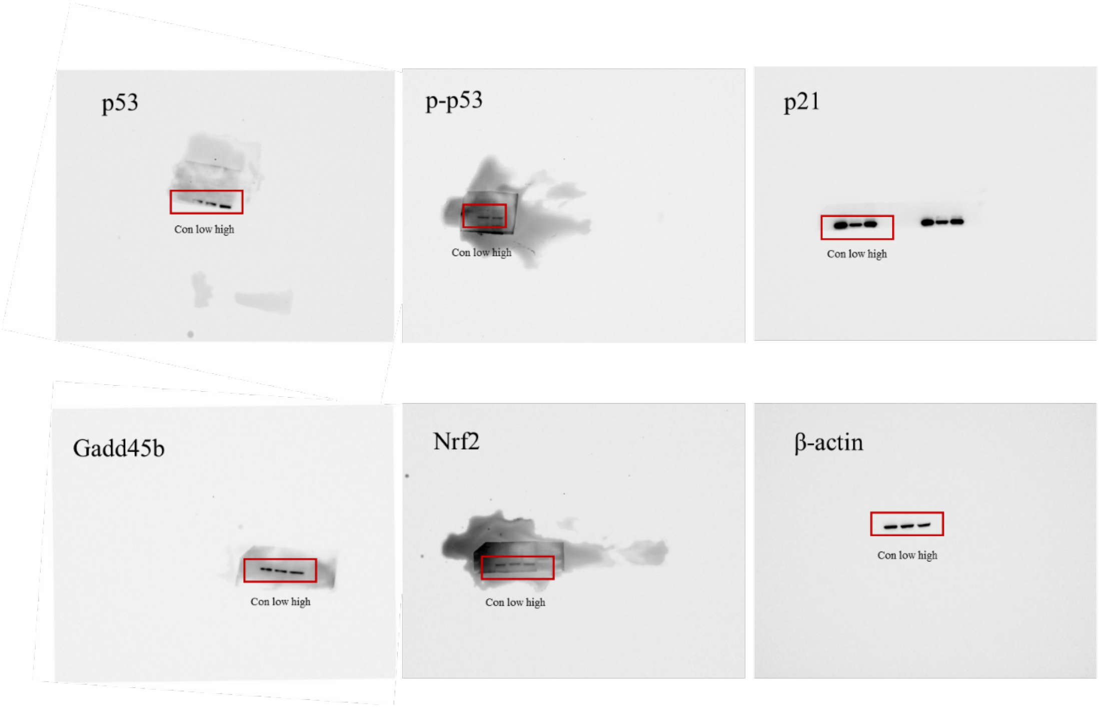

Figure S5. Full-length blots used to generate Figure 6a-total cell.

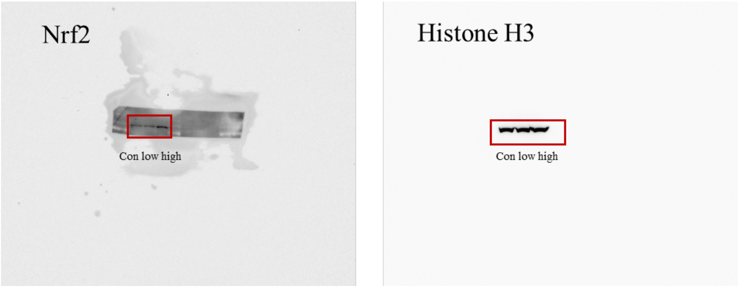

Figure S6. Full-length blots used to generate Figure 6a-nuclear.
